# Genome Sequences of Serotype A6 Mannheimia haemolytica Isolates D174 and D38 Recovered from Bovine Pneumonia

**DOI:** 10.1128/genomeA.00086-15

**Published:** 2015-03-05

**Authors:** Melissa J. Hauglund, Fred M. Tatum, Darrell O. Bayles, Samuel K. Maheswaran, Robert E. Briggs

**Affiliations:** a USDA, ARS, National Animal Disease Center, Ames, Iowa, USA; b Department of Veterinary and Biomedical Sciences, University of Minnesota, St. Paul, Minnesota, USA

## Abstract

Here, we report two genomes, one complete and one draft, from virulent bovine strains of Mannheimia haemolytica serotype A6 recovered prior to the field usage of modern antimicrobial drugs.

## GENOME ANNOUNCEMENT

Mannheimia haemolytica is a facultative respiratory pathogen of ruminants. Among cattle, serotypes A1, A2, and A6 colonize and are commonly recovered from the nasopharynx (1). In pneumonic disease, it is predominantly serotypes A1 and A6 that are recovered from diseased lung tissues (2). Resistance to nasopharyngeal colonization can be elicited with vaccine products, and such resistance has been shown to be serotype specific (3). Although the serotype of M. haemolytica is based on capsule type (2), it is currently unknown whether acquired resistance to nasopharyngeal colonization is based upon anticapsular or other bacterial components. Antimicrobial resistance among bacterial bovine respiratory disease pathogens is of growing concern (4, 5), and multidrug-resistant isolates of Pasteurella multocida and M. haemolytica were recently sequenced (6, 7). Isolates D174 and D38 were recovered from a pneumonic calf lung in January 1984 and December 1982, respectively. The genome sequencing of these strains was undertaken to further our understanding of the basis of acquired resistance to nasopharyngeal colonization and provide insight into the acquisition of antimicrobial resistance.

The genome sequencing of M. haemolytica strain D174 was achieved using 3 platforms: the Roche (454) GS FLX titanium, resulting in 25-fold coverage; Illumina GA IIx, resulting in 1,600-fold coverage; and PacBio RS, resulting in 21-fold coverage. The Illumina reads were used to error correct the PacBio reads using CLC Genomics Workbench version 6.0.2. A hybrid assembly using the CLC software was performed, and the resultant contigs were aligned to an optical map (MapSolver software; OpGen, Gaithersburg, MD) to confirm the assembly and generate a single scaffold. Reiterative alignments of the 454 and corrected PacBio reads >1 kb against the scaffold, using the CLC software, closed all gaps and resulted in a single circular chromosome. The completed D174 genome consists of 2.70 Mb, with a G+C content of 41.1%. The draft genome of M. haemolytica D38 was determined using the Roche platform alone, which yielded 24-fold coverage. Assembly against the closed D174 reference genome using the CLC software yielded 97 contigs with a total of 2.61 Mb, a G+C content of 41.0%, an *N*_50_ of 48,311 bp, and 100% contigs >500 bp.

The annotation of both genomes was accomplished with the NCBI Prokaryotic Genome Annotation Pipeline revision 2.1. Strain D174 contains a total of 2,814 genes, including 2,687 predicted protein-coding genes, 40 frameshifted pseudogenes, 19 rRNA genes, and 66 tRNA genes. Strain D38 contains a total of 2,710 genes, including 2,606 predicted protein-coding genes, 48 frameshifted pseudogenes, 6 rRNA genes, and 50 tRNA genes. One clustered regularly interspaced short palindromic repeat (CRISPR) array was detected in each isolate. In contrast to the multiresistant M. haemolytica isolate 42548 (6), genes *aphA1*, *strA*, *strB*, *sul2*, *tetR*, and *tetH* are absent in both strains D174 and D38. The determination of genes potentially involved serotype-specific resistance to nasopharyngeal colonization will require additional analysis.

### Nucleotide sequence accession numbers.

The genome sequence of M. haemolytica strain D174 has been deposited in GenBank under the accession no. CP006574. The genome sequence of M. haemolytica D38 has been deposited in GenBank under the accession no. AUNL00000000. The version described in this paper is version AUNL01000000.
